# The impact of COVID-19 on the lives of Canadians with and without non-communicable chronic diseases: results from the iCARE Study

**DOI:** 10.1186/s12889-023-15658-z

**Published:** 2023-10-26

**Authors:** Frédérique Deslauriers, Vincent Gosselin-Boucher, Camille Léger, Ariany Marques Vieira, Simon L. Bacon, Kim L. Lavoie

**Affiliations:** 1 Department of Psychology, Université du Québec à Montréal, Montreal, QC Canada; 2 Montreal Behavioural Medicine Centre, CIUSSS du Nord- de- l’Ile- de- Montreal, Montreal, Québec Canada; 3 School of Kinesiology, University of British Columbia, Vancouver, BC Canada; 4 Department of Health, Kinesiology and Applied Physiology, Concordia University, Montreal, QC Canada

**Keywords:** Non-communicable chronic disease, COVID-19, Impacts of the pandemic, Health behaviours, Mental health, Access to care, Sex inequalities

## Abstract

**Background:**

The COVID-19 pandemic and its prevention policies have taken a toll on Canadians, and certain subgroups may have been disproportionately affected, including those with non-communicable diseases (NCDs; e.g., heart and lung disease) due to their risk of COVID-19 complications and women due to excess domestic workload associated with traditional caregiver roles during the pandemic.

**Aims/Objectives:**

We investigated the impacts of COVID-19 on mental health, lifestyle habits, and access to healthcare among Canadians with NCDs compared to those without, and the extent to which women with NCDs were disproportionately affected.

**Methods:**

As part of the iCARE study (www.icarestudy.com), data from eight cross-sectional Canadian representative samples (total n = 24,028) was collected via online surveys between June 4, 2020 to February 2, 2022 and analyzed using general linear models.

**Results:**

A total of 45.6% (n = 10,570) of survey respondents indicated having at least one physician-diagnosed NCD, the most common of which were hypertension (24.3%), chronic lung disease (13.3%) and diabetes (12.0%). In fully adjusted models, those with NCDs were 1.18–1.24 times more likely to report feeling lonely, irritable/frustrated, and angry ‘to a great extent’ compared to those without (p’s < 0.001). Similarly, those with NCDs were 1.22–1.24 times more likely to report worse eating and drinking habits and cancelling medical appointments/avoiding the emergency department compared to those without (p’s < 0.001). Moreover, although there were no sex differences in access to medical care, women with NCDs were more likely to report feeling anxious and depressed, and report drinking less alcohol, compared to men with NCDs (p’s < 0.01).

**Conclusion:**

Results suggest that people with NCDs in general and women in general have been disproportionately more impacted by the pandemic, and that women with NCDs have suffered greater psychological distress (i.e., feeling anxious, depressed) compared to men, and men with NCDs reported having increased their alcohol consumption more since the start of COVID-19 compared to women. Findings point to potential intervention targets among people with NCDs (e.g., prioritizing access to medical care during a pandemic, increasing social support for this population and mental health support).

**Supplementary Information:**

The online version contains supplementary material available at 10.1186/s12889-023-15658-z.

## Background

The key to slowing the spread of COVID-19 has been widespread public adherence to the behaviour-based public health policies [1–3]. While the negative impacts of the pandemic have been felt worldwide, there is mounting evidence that they have not affected all segments of the population equally, identifying important impact disparities in specific groups that have been defined as vulnerable [4–8]. Among these groups includes people living with a non-communicable chronic disease (NCD; including cardiovascular diseases, chronic lung diseases, cancer, etc.), who have been shown to be at higher risk of COVID-19 morbidity, complications and death compared to those without NCDs [9–11].

Pre-pandemic, NCDs accounted for 71% of deaths worldwide [12]. In Canada, people living with NCDs represent 44% of the population aged 20 and over, and until the pandemic, treatment of NCDs accounted for the largest proportion of provincial healthcare costs [13]. NCDs are managed mostly through the receipt of routine care that includes various medical interventions (e.g., pharmacotherapy, various surgical and non-surgical interventions), engaging in good health behaviours, managing stress, and seeking urgent care when needed [14–16]. In Canada, many COVID-19 prevention policies resulted in the cancellation or suspension of “non urgent” medical care, including limiting access to allied healthcare professionals (e.g., nutritionists, kinesiologists) and rehabilitation programs important for supporting NCD patients to engage in good health behaviours [17]. General pandemic restrictions on movement and widespread closure of gyms and training facilities also reduced opportunities for NCD patients to maintain exercise regimens throughout the pandemic [17–19]. Indeed, there is evidence to suggest that adults suffering from various NCDs, including hypertension, chronic lung diseases, and obesity have been less physically active since the beginning of the pandemic compared to those without these conditions [19]. Lockdowns measures have also had an impact on drinking behaviour, which is also important for NCD risk [20, 21]. One study conducted in the UK found that alcohol consumption increased more among patients with NCDs during the pandemic compared to those without NCDs, which may increase their disease burden [20]. Although there is scattered evidence to suggest that people living with a NCD may have suffered some of the greatest direct and indirect impacts associated with the pandemic, little is known about the extent of these impacts in Canada, knowledge of which has implications for allocating treatment resources and future pandemic preparedness.

Another group who has been disproportionately impacted by the pandemic is women [22, 23]. The pandemic has intensified their levels of unpaid care and domestic workloads [24]. Studies have also shown that gender roles associated with child and elder care triggered by school closures and service gaps in assisted living homes have placed an excess burden upon women relative to men throughout the pandemic [8]. This excess burden has been linked to greater job disruptions and higher rates of unemployment in women compared to men [8, 23]. Occupations traditionally held by women were also among those hardest hit by the pandemic since women are disproportionately represented in front-line service occupations. Nearly 70% of the health, social services, and education workforce (e.g., nurses, community health workers, teachers) is occupied by women, all of whom faced significant daily health risks during the pandemic as a result of their occupation [23, 24]. Stay-at-home orders also had an impact on violence perpetrated against women, since it forced women to shelter in place with their abusers with few opportunities to seek out help [23]. It is therefore not surprising that the mental health toll of the pandemic was also disproportionately higher among women compared to men. Indeed, a recent systematic review showed that one of the most important risk factors for experiencing high levels of psychological distress during the pandemic was female sex; one of the others was the presence of an NCD [25]. Women account for at least 50% of patients living with NCDs (e.g., hypertension, asthma/COPD, cancer), yet what remains unknown is the impact of the pandemic on women vs. men living a NCD.

To address these research gaps, the primary objective of the present study was to assess how the COVID-19 pandemic has impacted Canadians with NCDs between June 4, 2020 and February 2, 2022 in three areas: mental health, health/lifestyle behaviours, and access to medical care. The secondary objective was to assess the extent to which women with NCDs were disproportionately affected relative to men across the same time period.

## Method

### Study design

The present study is a sub-analyses of the International COVID-19 Awareness and Responses Evaluation (iCARE) Study (https://www.icarestudy.com) led by researchers at the Montreal Behavioral Medicine Centre (MBMC: https://mbmc-cmcm.ca/).(26) This international, cross-sectional, multi-wave observational study aimed to examine public awareness, attitudes, and responses to COVID-19 public health policies through a series of online surveys. The impacts on diverse aspects of people’s lives since the start of the pandemic were also assessed. The study protocol and detailed methods have been published elsewhere [26].

### Sample

For this study, we analyzed data from eight cross-sectional Canadian representative samples (n = 3001 to 3006 each) collected between June 4, 2020 to February 2, 2022 (Timeline available in Supplementary Material, Figure S1). The total sample includes 24,028 Canadians aged 18 and over.

In order to collect reliable information on the Canadian population, samples were recruited through the proprietary online panel of Léger Opinion © (LégerWeb.com). This panel includes over 400,000 Canadians, the majority of whom (60%) were recruited within the past 10 years. Two thirds of the panel were recruited randomly by telephone, with the remainder recruited via publicity and social media. Using data from Statistics Canada, data were weighted within each province according to the sex and age of the respondents in order to make their profiles representative of the current population within each Canadian province (excluding the three territories). The weight of each province was further adjusted to represent their actual weight within the 10 Canadian provinces.

This study was approved by the Research Ethics Board of the Centre intégré universitaire de santé et de services sociaux du Nord-de-l’île-de-Montréal (CIUSSS-NIM), REB#: 2020–2099 / 25-03-2020.

### iCARE Survey

The survey included approximately 75 questions and took between 15 and 20 min to complete. The survey assessed a range of topics relevant to the pandemic including: (1) awareness of local government prevention policies to mitigate the spread of the virus (2) attitudes, perception, and adherence to these policies, (3) vaccine attitudes and intentions, (4) concerns about the virus, and (5) it’s impacts on several aspects of people’s lives (mental health, physical health, social relationships, employment and income, access to medical care). The survey also assessed several sociodemographic variables, as well as clinical and general health/mental health information, and health behaviors (e.g., diet, physical activity, substance use). The content of the survey was the same across surveys, with only minor adaptations to capture changes in the evolution of the pandemic over time. A detailed description and copy of all surveys can be found at the following weblink (https://osf.io/nswcm/?view_only=). For the present study, we extracted the following variables from the Canadian representative sample surveys 2 through 9 (June 3, 2020 to February 2, 2022, see Supplementary Figure S1): sociodemographic variables, chronic health conditions, and impacts of the pandemic. For more information on the list of measures available in the iCARE study as well as our data dictionaries, see https://osf.io/c5rux (accessed on 25 July 2022).

### Assessment of sociodemographic characteristics

The following sociodemographic characteristics were assessed across all eight Canadian surveys: sex, age, province, education level, average annual household income, current employment status, parental status, and prevalent mental health illness (i.e., depressive disorder, anxiety disorder). For the analyses, participants were categorized as man or woman depending on their self-reported answers to the following question: “How would you describe your sex? [Man, Woman, Other, I prefer not to answer]”.

### Assessment of NCDs

Participants self-reported having a diagnosis of one or more NCDs by answering the following question: “*To your knowledge, has a doctor or healthcare professional told you that you have any of the following health conditions? [Pulmonary disease, cardiac disease, obesity, cancer, diabetes, any autoimmune disease, any other condition that compromise the immune system, depressive disorder, anxiety disorder, Alzheimer’s and dementia]* (possible responses: yes, no, I don’t know/ prefer not to answer). Patients were classified as having an NCD if they responded yes to any of the above NCDs, excluding depressive disorders, anxiety disorders, or Alzheimer’s/dementia.

### Assessment of COVID-19 impacts

For this study, we report on data for the following three impact domains relevant to NCD risk and prognosis: mental health, health/lifestyle behaviours, and access to medical care [12, 27]. Each domain included several items, which have been summarized in Table 1. COVID-19 impacts on mental health and access to healthcare were assessed using the question: *“Please rate the extent to which COVID-19 has impacted the following aspects of your life over the last month”* with the following response options: ‘To a great extent, Somewhat, Very little, Not at all, I don’t know/I prefer not to answer, Not applicable*’.* COVID-19 impacts on health/lifestyle behaviours including, diet, physical activity, and alcohol consumption were measured using the question: *“In general, how have the following behaviours changed since the start of COVID-19?”* with the following response options: ‘I do this a lot more, I do this more, I do this as much as before, I do this less, I do this a lot less, I don’t do this, I don’t know/I prefer not to answer’.

**Table 1:** Impact domains and survey items

Domains of impact	Item of impact	*Survey Item*
**Mental Health**	Anxious	*I have felt nervous, anxious, or worried*
	Depressed	*I have felt sad, depressed, or hopeless*
	Lonely	*I have felt lonely and isolated*
	Irritable/frustrated/ angry	*I have felt irritable, frustrated or angry*
**Health/ lifestyle Behaviours**	Physical activity	*Doing physical activity*
	Diet	*Eating a healthy diet*
	Alcohol consumption	*Drinking alcohol*
**Access to care**	Avoided seeking medical care	*I have cancelled medical appointments or using hospital emergency services for a non-COVID-related problem*
	Access to non-COVID medical care	*I have had trouble getting non-COVID-19 related medical care*

### Statistical analyses

Several survey questions included an answer ‘I don’t know/I prefer not to answer’ which were recoded as missing values and analyses were conducted based on complete case records. Descriptive statistics (weighted means, SDs, and proportions) were calculated across all eight surveys to describe the sample in terms of demographic characteristics. Participants who reported at having least one physician diagnosis of an NCD (e.g.: cardiovascular disease, chronic lung disease, obesity, cancer, diabetes, any autoimmune disease, or any other condition that compromises the immune system) were classified as having an NCD. Bivariate analyses were conducted to examine differences in sociodemographic characteristics (weighted proportions) as a function of NCD status as well as NCD status and sex across the eight surveys. The frequencies of participants that reported being impacted “to a great extent” (compared to ‘somewhat, very little and not at all’) for each impact item were calculated as a function of NCD status and sex using chi-square tests. For the health behaviours, the responses were dichotomized into two groups: those who engaged in a positive health behaviour (e.g., ate a healthy diet, engaged in physical activity) ‘a lot less or less’, compared to those who engaged in it ‘as much as before, more, or much more’, and those who engaged in a negative health behaviour (e.g., alcohol use) ‘a lot more or more’ compared to ‘the same as before, less or a lot less’.

In order to assess the impacts of COVID-19 in patients with vs. without NCDs as a function of sex, we conducted a series of multivariable models for each impact item. Each model included the interaction of NCD and sex. For each impact, two models were run: Model 1 examined the main effects of NCD and sex while adjusting for covariates (sex, age, education, annual income, employment status, survey round, presence of mental health) chosen a-priori based on established associations between them and NCDs, sex and/or impact outcome variables [18, 28–30]. Model 2 examined the main and interaction effects of NCD and sex and was also adjusted for the same covariates as model 1. We analyzed weighted data for all the models. All statistical tests were two-tailed and p < .05 was considered as statistically significant. All analysis was performed in SAS version 9.4.

## Results

### Sample description

The total sample included 24,028 individuals, and just over half were female (51.8%) with a mean age of 48.30 (SD = 16.94), and most had an education level of high school or lower (67.5%). A total of 46% reported having one or more physician-diagnosed NCD (n = 10,570) across the eight surveys, the most common of which were hypertension (24.3%), chronic lung disease (13.3%) and diabetes (12%) (for a complete list of the frequencies of various NCDs, see Table 2).

**Table 2:** Distribution of non-communicable chronic diseases across the sample (n = 24,028)

Non-communicable chronic diseases	% (n)
Hypertension	24.3 (5701)
Any chronic lung disease (e.g., asthma, COPD)	13.3 (3127)
Diabetes	12 (2811)
Any autoimmune disease (e.g., rheumatoid arthritis)	9.8 (2284)
Any cardiovascular disease (e.g., heart disease, stroke)	9.0 (2121)
Obesity	7.7 (1 814)
Other condition that compromises the immunes system (e.g., organ transplant)	4.6 (1079)
Active/current cancer	2.8 (668)

Sample characteristics presented as function of NCD status are presented in Table 3. Compared to participants without NCDs, those with NCDs were older (NCD M age = 54.75 years [SD = 16.15]; non-NCD M age = 43.66 years [SD = 15.91]) (p **<** .001), had a high school education or less (NCD: 71.3; non-NCD: 63.7%, p < .001), had an annual household income under $60,000/year (NCD: 51.5%; non-NCD: 41.5%, p < .001), and were more likely to report having a mental health disorder (NCD: 34.5%; non-NCD 19.1%, p < .001).

**Table 3:** Participant characteristics as a function of NCD status

Descriptive characteristics	No NCD (n = 12 601)	NCD (n = 10 570)	p-value
Variable	% (n)	% (n)	
**Sex**			
*Man*	48.7 (6 095)	47.9 (5 042)	0.243
*Woman*	51.3 (6 426)	52.1 (5 483)	
*Missing values*	983		
**Age**			
*≥ 25 years*	17.3 (2 153)	5.8 (609)	**< .001**
*26–50 years*	64.1 (5 922)	31.6 (3 321)	
*≥ 51 years*	39.9 (4 360)	62.6 (6 566)	
*Missing values*	1 094		
*Mean (SD)*	43.7 (15.9)	54.7 (16).1	
**Education level**			
*High school or lower*	63.9 (7 952)	71.3 (7 442)	**< .001**
*Graduate or Postgraduate degree*	36.2 (4 502)	28.7 (3 002)	
*Missing values*	1 127		
**Region (% from each province)**			
*British Columbia*	13.6 (1710)	13.2 (1397)	**< .001**
*Alberta*	11.3 (1424)	11.0 (1158)	
*Saskatchewan*	2.6 (322)	3.1 (324)	
*Manitoba*	3.4 (429)	4.0 (422)	
*Ontario*	38.3 (4820)	38.6 (4078)	
*Quebec*	24.7 (3114)	22.5 (2374)	
*New Brunswick*	2.1 (269)	2.5 (264)	
*Nova Scotia*	2.5 (320)	3.2 (336)	
*Prince Edward Island*	0.3 (43)	0.3 (32.1)	
*Newfoundland*	1.2 (151)	1.8 (185)	
*Missing values*	857		
**Currently employed**			
*Yes*	60.8 (7 560)	43.2 (4 497)	**< .001**
*Missing values*	1 184		
**Average annual household income**			
*< 60 000 $/year*	41.5 (4 654)	51.5 (4 895)	**< .001**
*≥ 60 000$/year*	58.5 (6 565)	48.5 (4 617)	
*Missing values*	3 298		
**Presence of any mental health disorder (e.g., depression, anxiety)**			
*Yes*	19.1 (2 376)	34.5 (3 574)	**< .001**
*Missing values*	1218		
**Presence of any depressive disorder**			
*Yes*	11.4 (1 421)	23.1 (2 399)	**< .001**
*Missing values*	1162		
**Presence of any anxiety disorder**			
*Yes*	16.4 (2 043)	28.5 (2 960)	**< .001**
*Missing values*	1158		
**Essential service worker**			
*Yes*	37.3 (2 747)	40.3 (1 782)	**0.001**
*Missing values*	12 247		
**Health care worker**			
*Yes*	25.6 (674)	22.0 (389)	**0.044**
*Missing values*	19 523		
**History of COVID-19 infection**			
*COVID-19 positive*	6.4 (109)	5.0 (88)	**0.001**
*COVID-19 negative*	92.9 (1 604)	92.8 (1 630)	
*I am still waiting for my result*	0.7 (12)	2.2 (39)	
*Missing values*	20 545		

NCD, non-communicable chronic disease

We also presented sample characteristics in those with NCDs as a function of sex in Supplementary Table S1. In general, compared to men with an NCD, women with an NCD were younger, had a high school education or less, and were more likely to be unemployed, have an annual household income under $60,000/year, and report having a mental health disorder (all p’s < 0.001).

### Impacts of COVID-19 in respondents with versus without an NCD

The results of multivariable analyses presenting the OR (95% CI) of reporting each impact ‘to a great extent’ as a function of NCD status adjusting for sex, age, education, annual income, employment status, survey round and presence of a mental health disorder can be found in Table 4. The results showed that compared to those without an NCD, those with an NCD were significantly more likely to have been impacted across all variables except for self-reported increased alcohol consumption since the start of COVID-19. Specifically, those with an NCD were 21% more likely to report significant anxiety (OR 1.21, 95%CI 1.11–1.31), depression (OR 1.19, 95%CI 1.09–1.30), loneliness (OR 1.24, 95% CI 1.14–1.35), and anger (OR 1.18, 95% CI 1.09–1.29). Respondents with an NCD were also 24% and 22% more likely to report doing less physical activity (OR 1.24, 95%CI 1.15–1.33) and eating a healthy diet (OR 1.22, 95% CI 1.12–1.33) ‘less/a lot less’, respectively, compared to those without an NCD. Finally, those with an NCD were 57% more likely to report cancelling medical appointments or avoiding going to the emergency department (OR 1.57, 95%CI 1.42–1.74) and 87% more likely to report having trouble accessing non-COVID-19-related medical care (OR 1.87, 95% CI 1.68–2.08) ‘to a great extent’ compared to those without an NCD.

**Table 4:** Adjusted* Odds ratio of reporting each impact “to a great extent“ as a function NCD status

	People with at least one NCD) (Reference: people with no NCD)				
	OR	95% CI			
		Lower	Upper	p-value	
**Mental health**					
Anxious	1.21	1.11	1.31	**< .001**	
Depressed	1.19	1.09	1.30	**< .001**	
Lonely	1.24	1.14	1.35	**< .001**	
Irritable/frustrated/angry	1.18	1.09	1.29	**< .001**	
**Health/lifestyle behaviours**					
Less physically active	1.24	1.15	1.33	**< .001**	
Worse diet	1.22	1.12	1.33	**< .001**	
Increased alcohol consumption	1.03	0.93	1.13	0.690	
**Access to care**					
I have cancelled medical appointments or using hospital emergency services for a non-COVID-related problem	1.57	1.42	1.74	**< .001**	
I have had trouble getting non-COVID-19 related medical care	1.87	1.68	2.08	**< .001**	

OR, Odds Ratio; CI, Confidence interval; NCD, non-communicable chronic disease

We also re-ran our adjusted multivariable analyses of reporting each impact ‘to a great extent’ as a function of sex rather than NCD status, which can be found in Supplementary Table S2. With the exception of having trouble accessing non-COVID-19-related medical care, women in general were significantly more likely to have been impacted across all mental health, health behaviour and access to care variables compared to men (all p’s < 0.001).

### Impacts of COVID-19 in NCD patients as a function of sex

The results of multivariable analyses adjusting for sex, age, education, annual income, employment status, survey round and presence of mental health condition showing the OR (95% CI) of reporting each impact ‘to a great extent’ as a function of NCD and sex can be found in Table 5. The results revealed significant interactions between NCD status and sex for three impact variables: “anxious”, “depressed” and self-reported “increased alcohol consumption since the start of COVID-19”. As can be seen Table 5, women with an NCD were more likely to report feeling anxious ‘to a great extent’ compared to men with an NCD (OR 1.61, 95% CI 1.44–1.81), women without an NCD were more likely to report feeling anxious ‘to a great extent’ compared to men without an NCD (OR 2.04, 95% CI 1.83–2.27), and men with an NCD were more likely to report feeling anxious ‘to a great extent’ compared to men without an NCD (OR 1.23, 95% CI 1.23–1.58) (p = .004). Similar trends were observed for depression (p < .01, see Table 5).

**Table 5:** Adjusted* odds ratio of reporting each impact ?to a great extent? as a function of the NCD status and sex interaction

	p-value for the interaction NCD & sex		Women with NCD (Reference: Women with no NCD)			Men with NCD (Reference: Men with no NCD)			Women with NCD (Reference: Men with NCD)			Women with no NCD (Reference: Men with no NCD)		
			OR	95% CI		OR	95% CI		OR	95% CI		OR	95% CI	
				Lower	Upper		Lower	Upper		Lower	Upper		Lower	Upper
**Mental health**														
Anxious	**.004**		1.10	0.99	1.22	**1.39**	**1.23**	**1.58**	**1.61**	**1.44**	**1.81**	**2.04**	**1.83**	**2.27**
Depressed	**.010**		1.09	0.97	1.22	**1.35**	**1.18**	**1.53**	**1.26**	**1.12**	**1.42**	**1.57**	**1.40**	**1.76**
Lonely	.086		1.17	1.05	1.30	1.34	1.18	1.52	1.37	1.22	1.53	1.57	1.41	1.75
Irritable/ frustrated or angry	.327		1.14	1.03	1.27	1.24	1.09	1.40	1.35	1.20	1.52	1.46	1.31	1.63
**Health/lifestyle behaviours**														
Less physically active	.195		1.19	1.08	1.31	1.30	1.17	1.44	1.15	1.05	1.27	1.262	1.15	1.39
Worse diet	.578		1.20	1.07	1.34	1.25	1.12	1.42	1.25	1.11	1.41	1.31	1.17	1.47
Increased alcohol consumption	**< .001**		**0.84**	**0.74**	**0.96**	**1.23**	**1.09**	**1.40**	**0.69**	**0.61**	**0.79**	1.01	0.90	1.14
**Access to care**														
I cancelled medical appointments or avoided presenting to the emergency department	.595		1.54	1.353	1.75	1.62	1.40	1.87	1.23	1.08	1.39	1.29	1.12	1.49
I had trouble getting access to non-COVID medical care	.971		1.86	1.62	2.15	1.87	1.61	2.17	1.00	0.88	1.13	1.00	0.86	1.16

OR, Odds Ratio; CI, Confidence interval; NCD, non-communicable chronic disease

*Adjusted for for sex, age, education, annual income, employment status, survey round and presence of mental health

Finally, women with an NCD were less likely to report increasing their alcohol consumption since the start of COVID-19 compared to women without a NCD (OR 0.84, 95% CI 0.74–0.96), women with an NCD were less likely to report increasing their alcohol consumption since the start of COVID-19 compared to men with an NCD (OR 0.61, 95% CI 0.61–0.79), and men with an NCD were more likely to report increasing their alcohol consumption since the start of COVID-19 compared to men without an NCD (OR 1.23, 95% CI 1.09–1.40) (p < .001). There was no significant NCD x sex interactions on any access to care variables.

## Discussion

The results of this study show that between the June 4, 2020 and February 2, 2022, which covers the first two peaks of the pandemic in Canada, people with an NCD were disproportionally more impacted by the pandemic in terms of impacts on mental health, health behaviours and access to medical care compared to Canadians without a NCD. Specifically, Canadians living with an NCD tended to cancel more of their medical appointments and avoid going to the emergency than people without an NCD. These findings are consistent with a study outside Canada looking at delayed and cancelled healthcare during COVID-19, which reports that three-quarters of adults whose healthcare was delayed or cancelled suffered from one or more NCD [31]. There are several plausible explanations for this, including the possibility that NCD patients, by virtue of having one or more chronic condition, may have had more medical appointments to begin with, making them more likely to have an appointment to cancel relative to those without a chronic health condition. It is also possible that since suffering from an NCD is an important risk factor for COVID-19 complications, people with an NCD were more likely to cancel their medical appointments for fear of exposing themselves to the virus in the hospital or clinic setting. This is supported by a recent study showing that nearly 41% of Americans said they had delayed or avoided all medical care, including urgent care (12%) and disease management care (31.5%), due to concerns about COVID-19, [32] a finding that is likely accentuated among those at higher baseline risk. This is consistent with other research highlighting the important role of fear of contracting COVID-19 in decisions about whether or not to cancel medical appointments [33, 34]. Interestingly, patients with an NCD also reported having more difficulty accessing non-COVID-19-related medical care, which represents a distinct subgroup who actively sought out care that was not available. This is likely due to healthcare resource shortages that were prevalent over the course of the study period due to excess demands placed on healthcare systems to treat COVID-19 patients [33].

Consistent with this is a WHO survey that reported the suspension of chronic disease rehabilitation programs (e.g., cardiopulmonary) among 81% of participating countries, which are among the first-line treatments for patients with NCDs [33]. Taken together, these results point to important treatment gaps among people living with NCDs over the course of the pandemic.

In addition to having less access to care, people with an NCD also reported significant negative impacts on their eating behaviour and levels of physical activity compared to those without, behaviours which are important for disease management among those with NCDs. These findings are consistent with a study by Rogers et al., who also observed worse health behaviours in those with NCDs vs. without over the course of the pandemic [19]. This finding may be explained by having fewer access to allied healthcare provider support (e.g., nutritionists, kinesiologists), reduced access to healthy foods or recreational facilities (e.g., gyms) due to closures and movement restrictions, or decreased motivation to maintain good health behaviours during the pandemic [35].

When comparing men to women, we also observed that with the exception of worse access to medical care (which appears to have been uniformly reduced), women were generally more impacted across all impact domains compared to men. In terms of mental health, women were more likely to report suffering from severe anxiety, depression, isolation and anger than men, which is consistent with the results of previous studies [22, 25]. This may be explained in part by the fact that 31% of women in our study reported having a comorbid mental health disorder (i.e., anxiety or depressive disorder) compared to only 20% of men. Also, more women in our sample were healthcare workers, who experienced some of the highest rates of stress over the course of the pandemic due to their essential worker status and high risk of virus exposure [36, 37]. Women may have experienced greater mental health impacts compared to men due to gender roles linked to employment status, lack of social support, parenthood, and caregiving responsibilities [22]. These gender roles may have increased women’s risk of experiencing psychological distress since they were more likely to spend time at home, assume childcare responsibilities, and care for older family members [8]. According to data from previous pandemics, women with caregiving responsibilities and resource insecurity are at greater risk of developing mental health disorders [22]. A multinational survey also found that women lacking in social support are more vulnerable to negative mental health impacts compared to men, due to women’s greater reliance on social support [22]. Since people were unable to access their social support network during the pandemic due to movement restrictions, women may have felt the negative effects of isolation more acutely [22].

Although people with versus without NCDs and women versus men were generally more impacted by the pandemic, we did observe some interesting sex by NCD interactions. Specifically, women with NCDs experienced worse mental health impacts (anxiety, depression) compared to men with NCDs, men with NCDs experienced worse mental health impacts (anxiety, depression) and greater increases in alcohol consumption than men without NCDs, and women with NCDs experienced lower increases in alcohol consumption compared to women without NCDs. Mental health results are consistent with a study conducted on Bangladeshi adults which also reported worse symptoms of anxiety and depression in women with an NCD compared to men, [38] and points to a need to provide greater mental health support among women with NCDs, which may important in light of important impacts of anxiety and depression on NCD outcomes [39]. However, our alcohol consumption results are not entirely consistent with those of previous studies, which have generally reported greater increases in alcohol consumption among women compared to men during the pandemic [40, 41]. One explanation for the inconsistent findings is that previous studies did not look at changes in drinking behaviour as a function of NCD status. Given that women tend to be more conscientious about their health than men, it is possible that women with NCDs were less likely to turn to alcohol during the pandemic due to their negative effects on health, compared to men [42].

### Limitations and strengths

This study has certain limitations that may influence the interpretation of the results. First, data was self-reported and may be subject to social desirability and reporting bias. Second, the results only reflect the impacts of the first 18 months of the pandemic (during the first two waves), and impacts may have decreased over time. Third, NCD status was self-reported and not objectively verified. Though we asked people to self-report on physician-diagnosed conditions, it is still possible that some people might have misinterpreted their diagnosis. Moreover, we only reported on the presence and not on the severity of chronic conditions, which was beyond the scope of the survey. It is therefore not possible to determine whether people with a more serious health condition were more affected by COVID-19. Finally, we only asked about the extent to which people’s mental health and health behaviours were affected by the pandemic, we did not administer validated psychological questionnaires or directly measure health behaviours, which was also beyond the scope of the study.

Despite some limitations, this study also has several strengths. The data collection period makes it possible to assess the effects of the pandemic over the first two waves of the pandemic in Canada. The study included a large sample size, respondents were well distributed across provincial regions, age groups, employment status and income compared to census data available through Statistics Canada, and there were equal proportions of men and women which facilitated analyses by sex. Assessing a range of impacts made it possible to estimate the overall impact of the pandemic on vulnerable populations. Finally, results reflect a sub-analysis of Canadian representative data from the iCARE study, which has collected data from more than 130,000 people from 190 countries to date (see: www.icarestudy.com). This will facilitate comparisons with international datasets to contribute important evidence syntheses on the impacts of the pandemic on vulnerable groups that can support intervention and policy strategies worldwide.

## Conclusions and future perspective

Results suggest that people with NCDs in general and women in general have been disproportionately more impacted by the pandemic, and that women with NCDs have suffered greater psychological distress (i.e., feeling anxious, depressed) compared to men, and men with NCDs self-reported having increased more their alcohol consumption since the start of COVID-19 compared to women. Findings add to the body of literature showing important disparities in pandemic impacts among vulnerable groups, supporting the need for greater resources and interventions in these populations to mitigate these impacts of the current pandemic and prevent these impacts in future ones.

### Electronic supplementary material

Below is the link to the electronic supplementary material.

Supplementary Material 1

## Data Availability

The datasets generated and/or analysed during the current study are not publicly available in order to comply with institutional ethics approvals, but can be made available by contacting the corresponding autho​r on reasonable request OR accessed directly by requesting raw data through our data access process (found at https://mbmc-cmcm.ca/covid19/apl/).

## List of Abbreviations

COPD: Chronic Obstructive Pulmonary Disease
COVID-19: Corona Virus Disease 19
iCARE: International COVID-19 Awareness and Responses Evaluation
NCD: non-communicable chronic disease
