# Supplementary material for: The impact of COVID-19 on the lives of Canadians with and without non-communicable chronic diseases: results from the iCARE Study
Source: BMC Public Health. 2023 Oct 26;23:2106. doi: 10.1186/s12889-023-15658-z (PMC10604733; doi:10.1186/s12889-023-15658-z)
Supplement: Supplementary file 1 — Supplementary Material 1 [file 12889_2023_15658_MOESM1_ESM.docx]

**Supplementary file**

**Figure S1**

*Dates and periods of the 8 Canadian surveys*

*
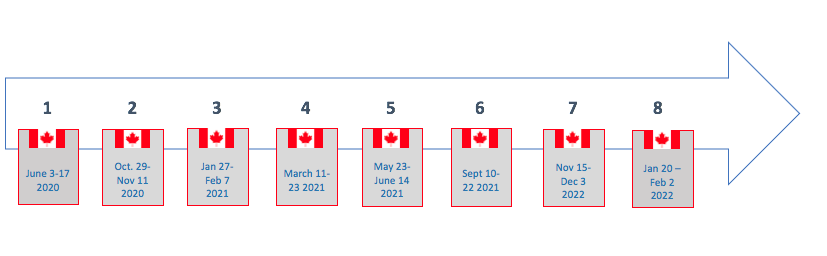
*

**Table S1**

*Participant characteristics as a function of NCD status and sex*

| **Descriptive characteristics** | **Men with NCD**  **(n= 5 042)** | | | **Women with NCD**  **(n=5 483)** | | |  | |  |
| --- | --- | --- | --- | --- | --- | --- | --- | --- | --- |
| **Variable** | **% (n)** | | | **% (n)** | | | **p-value** | |  |
| **Age** |  | | |  | | |  | |  |
| *≤25 years* | 5.96 (298) | | | 5.39 (293) | | | **0.001** | |  |
| *26-50 years* | 26.6 (1 484) | | | 33.4 (1 817) | | |  | |  |
| *≥51 years or more* | 64.4 (3 228) | | | 61.2 (3 330) | | |  | |  |
| *Missing values* | 119 | | |  | | |  | |  |
| *Mean (SD)* | 56.7 (15.6) | | | 52.8 (16.3) | | |  | |  |
| **Education level** |  | | |  | | |  | |  |
| *High school or lower* | 70.0 (3 484) | | | 72.3 (3 932) | | | **0.011** | |  |
| *Graduate or Postgraduate degree* | 29.9 (1 489) | | | 27.6 (1 506) | | |  | |  |
| *Missing values* | 160 | | |  | | |  | |  |
| **Region (% from each province)** | | | | | | | | |  |
| *British Columbia* | 13.5 (680) | | | 13.0 (710) | | | 0.733 | |  |
| *Alberta* | 10.7 (584) | | | 10.7 (584) | | |  | |  |
| *Saskatchewan* | 3.1 (168) | | | 3.1 (168) | | |  | |  |
| *Manitoba* | 4.1 (226) | | | 4.1 (226) | | |  | |  |
| *Ontario* | 38.5 (2110) | | | 38.5 (2110) | | |  | |  |
| *Quebec* | 22.6 (1238) | | | 22.6 (1238) | | |  | |  |
| *New Brunswick* | 2.7 (146) | | | 2.7 (146) | | |  | |  |
| *Nova Scotia* | 3.2 (176) | | | 3.2 (176) | | |  | |  |
| *Prince Edward Island* | 0.3 (15.8) | | | 0.3 (16) | | |  | |  |
| *Newfoundland* | 2.0 (108) | | | 2.0 (108) | | |  | |  |
| *Missing values* | 46 | | |  | | |  | |  |
| **Currently employed** | | | | | | | | |  |
| *Yes* | 46.4 (2 297) | | | 40.4 (2 186) | | | **<.001** | |  |
| *Missing values* | 209 | | |  | | |  | |  |
| **Average annual household income** | | | | | | | | |  |
| *<$60 000/year* | 46.6 (2 193) | | | 56.1 (2 672) | | | **<.001** | |  |
| *≥$60 000/year* | 53.4 (2 516) | | | 43.9 (2 094) | | |  | |  |
| *Missing values* | 1095 | | |  | | |  | |  |
| **Presence of any mental health disorder (e.g., depression, anxiety)** | | | | | | | | |  |
| *No* | 71.2 (3 531) | | | 60.8 (3 263) | | |  | |  |
| *Yes* | 28.8 (1 427) | | | 39.2 (2 107) | | | **<.001** | |  |
| *Missing values* | 241 | | |  | | |  | |  |
| **Presence of any depressive disorder** | | |  |  |  | | |  | |
| *No* | 80.2 (3 983) | | | 74.2 (3 992) | | | **<.001** | |  |
| *Yes* | 19.8 (984) | | | 25.8 (1 387) | | |  | |  |
| *Missing values* | 222 | | |  | | |  | |  |
| **Presence of any anxiety disorder** | |  | |  |  |  |  |  |  |
| *No* | 77.3 (3 843) | | | 66.7 (3 591) | | | **<.001** | |  |
| *Yes* | 22.7 (1 129) | | | 33.3 (1 791) | | |  | |  |
| *Missing values* | 216 | | |  | | |  | |  |
| **History of COVID-19 infection** | | | | | | | | |  |
| *COVID-19 positive* | 5.1 (43) | | | 5.1 (45) | | | 0.091 | |  |
| *COVID-19 negative* | 91.9 (784) | | | 93.5 (830) | | |  | |  |
| *I am still waiting for my result* | 3.1 (26) | | | 1.5 (13) | | |  | |  |
| *Missing values* | 8 829 | | |  | | |  | |  |

NCD: non-communicable chronic disease

**Table S2**

*Frequency of reporting each impact “to a great extent” as a function of NCD status and sex*

|  | No NCD  (n=12 601) | | NCD  (n=10 570) | | | |  |
| --- | --- | --- | --- | --- | --- | --- | --- |
|  | Men  (n=6095) | Women  (n=6426) | Men  (n=5042)  % (n) | | Women  (n=5483)  % (n) | | |
|  | % (n) | % (n) |  |  |  |  |  |
| **Mental health** | | | | | | |  |
| Anxious | 12.48 (741) | 24.33 (1538) | 16.03 (789) | 26.20 (1402) | | |  |
| Depressed | 11.88 (705) | 19.74 (1241) | 14.9 (731) | 21.25 (1140) | | |  |
| Lonely | 13.38 (791) | 21.22 (1334) | 16.03 (784) | 23.38 (1252) | | |  |
| Irritable/ frustrated or angry | 13.52 (802) | 20.02 (1253) | 14.73 (725) | 21.02 (1123) | | |  |
| **Health behaviours** |  |  |  |  | |  |  |
| Less physically active | 31.33 (1402) | 36.75 (1731) | 35.18 (1336) | 39.64 (1641) | |  |  |
| Worse diet | 18.09 (809) | 22.92 (1076) | 19.69 (744) | 24.36 (1011) | |  |  |
| Increased alcohol consumption | 17.95 (803) | 17.67 (829) | 18.02 (684) | 14.19 (586) | |  |  |
| **Access to care** |  |  |  |  | |  |  |
| I cancelled medical appointments or avoided presenting to the emergency department | 8.85 (456) | 11.76 (634) | 13.00 (583) | 18.21 (870) | |  |  |
| I had trouble getting access to non-COVID medical care | 8.40 (429) | 9.39 (503) | 13.07 (586) | 14.89 (713) | |  |  |

NCD: non-communicable chronic disease

**Table S3**

*Adjusted* odds ratio of reporting each impact “to a great extent” as a function of sex*

|  | **Women**  **(Reference: Men)** | | | | | | | | | |  |
| --- | --- | --- | --- | --- | --- | --- | --- | --- | --- | --- | --- |
|  | OR | 95% CI | | | | | | | |  |  |
|  |  | Lower | | | | | Upper | | | p-value |  |
| **Mental health** |  | | |  | | | |  | | |  |
| Anxious | 1.820 | | 1.68 | | | | 1.97 | | | **<.001** |  |
| Depressed | 1.411 | | 1.30 | | | | 1.53 | | | **<.001** |  |
| Lonely | 1.47 | | 1.36 | | | | 1.59 | | | **<.001** |  |
| Irritable/frustrated/angry | 1.41 | | 1.30 | | | | 1.53 | | | **<.001** |  |
| **Health behaviours** |  | | |  | | | |  | | |  |
| Less physically active | 1.21 | | 1.13 | | 1.30 | | | | **<.001** | |  |
| Worse diet | 1.28 | | 1.18 | | 1.39 | | | | **<.001** | |  |
| Increased alcohol consumption | 0.86 | | 0.79 | | 0.94 | | | | **<.001** | |  |
| **Access to care** |  | | |  | | | |  | | |  |
| I cancelled medical appointments or avoided presenting to the emergency department | 1.26 | | 1.14 | | | 1.38 | | | | **<.001** |  |
| I had trouble getting access to non-COVID medical care | 0.99 | | 0.90 | | | 1.10 | | | | .972 |  |

OR: Odds Ratio, CI: Confidence interval, NCD: non-communicable chronic disease.

*Adjusted for sex, age, education, annual income, employment status, survey round and presence of mental health
